# Evaluation of the effects of diode laser application on experimental orthodontic tooth movements in rats. Histopathological analysis^1^

**DOI:** 10.1590/ACB351204

**Published:** 2021-01-20

**Authors:** Mehmet Ali Karabel, Mehmet Doğru, Arzum Doğru, Mehmet İrfan Karadede, Mehmet Cudi Tuncer

**Affiliations:** I PhD, Specialist of Orthodontics, Turkey.; II PhD, Assistant Professor, Department of Orthodontics, Faculty of Dentistry, Dicle University, Diyarbakır, Turkey.; III PhD, Associate Professor, Department of Periodontology, Faculty of Dentistry, Dicle University, Diyarbakır, Turkey.; IV PhD, Department of Orthodontics, Faculty of Dentistry, İzmir Katip Çelebi University, İzmir, Turkey.; V PhD, Professor, Department of Anatomy, Faculty of Medicine, Dicle University, Diyarbakır, Turkey.

**Keywords:** Tooth Movement, Techniques, Lasers, Semiconductor, Rats

## Abstract

**Purpose:**

To evaluate the effect of diode laser use on experimental orthodontic tooth movements.

**Methods:**

Thirty *Rattus norvegicus albinus* Wistar were divided into three equal groups (n = 10), two experimentals and one control. Applying 20 g orthodontic force were attached to the maxillary incisors of the rats in all groups. Low dose laser was applied to the surrounding tissues of the maxillary incisors of the rats in the experimental groups. Two exposure times for laser irradiation were used for seven days: t = 12 min (energy dose = 72 J) and t = 9 min (energy dose = 54 J) by a 0.1 W DEKA brand diode laser with wavelength of 980 nm.

**Results:**

Osteoclastic activation increased in the 72 J group when compared to control group and decreased in comparison to the 54 J group. Osteoblastic activation was decreased in the 72 J group when compared to the control group and increased in comparison to the 54 J group.

**Conclusions:**

Applying 54 J laser energy has been found effective to accelerate the orthodontic tooth movement.

## Introduction

Orthodontics is the science that aims to treat malocclusion and anomalies in the teeth, jaw and face by applying orthodontic forces and obtaining an optimal aesthetic and function. A good patient cooperation is extremely important for the success of the treatment. Goulart *et al*.^1^ reported that the main complaint about orthodontic treatment is the duration of treatment. Although orthodontics is one of the oldest known treatment methods in dentistry, it is still among the longest-lasting treatments. Optimum force is applied within physiological limits during orthodontic treatment. While direct bone resorption can be achieved with optimal forces, increasing the force level to accelerate orthodontic tooth movement causes the blood flow to stop in the periodontal ligament in the pressure zone and indirect bone resorption as a result of the development of pathological process called hyalinization; this slows down orthodontic tooth movements^2^–^5^.

The treatment of orthodontic anomalies is the movement of teeth from one place to another within the alveolar bone as a result of some biological processes occurring in the periodonium with the optimum force mechanics applied. The basis of the movement of a tooth in the alveolar bone is the remodeling of the alveolar bone that surrounds the root of that tooth and some cellular changes in the periodontal ligament. In an experimental orthodontic study on dogs in 1904, it was reported that there was a compression in the periodontal ligament in the direction of motion of the tooth, followed by a resorption in the alveolar bone in the direction in which the tooth was moving and in the opposite direction of the motion. As a result of the tension of the periodontal ligament, it was enlarged and occured apposition of the alveolar bone (bone formation)^6^. In this period, the alveolar bone is reshaped and tooth movement occurs in the direction of force. The slowness of this process is caused by bone turnover and remodeling, which can cause to poor patient cooperation and affect treatments negatively.

Many scientific studies have been done to solve this problem. For this purpose, many pharmacological agents have been used to accelerate orthodontic tooth movements, such as acetyl salicylic acid, dihydroxycholecalciferol^7^^,^ 8, nitric oxide^9^, thyroid hormones^10^, cortisone^11^, prostaglandins^12^, inflammatory cytokines^13^, osteocalcin^14^. In addition, orthodontic tooth movement was tried to accelerate by mechanical applications, such as mechanical vibration^15^, electrical current towards mechanical forces, pulsed electromagnetic field^16^–^18^, corticotomy^19^ and laser applications^19^–^22^ that have become widespread and developed in recent years.

Studies investigating the biostimulative effect of laser on orthodontics and tooth movement have started to increase in order to shorten the long-term treatment process and to strengthen the reduced patient due to the slow remodeling process of the bone in orthodontic treatments. In this way, the attempt is to increase physician cooperation and patients desire for orthodontic treatment^19^–^29^. The mechanism of action of low-dose laser therapies work with the capacity of subcellular photoreceptors to respond to visible red and infrared wavelengths. Changes in the enzymatic and photochemical activities of tissues occur with the stimulation of these receptors^30^^,^ 31. Electron transport chain, respiratory cycle and oxidation mechanisms are affected and an increase in cellular metabolic processes occurs^32^. Histomorphological effects have been demonstrated in studies conducted, such as increased fibroblastic (fibroblast proliferation, collagen secretion, collagen matrix deposition), osteoblastic and osteoclastic activities and bone resorption and acceleration of the alveolar bone cycle^30^^,^ 33 ^,^ 34.

However, although the studies on this subject are insufficient, there is no consensus on the way the laser is applied, its dosage, the way it creates orthodontic tooth movement and whether it increases tooth movement.

This study was aimed to evaluate the effect of low-dose diode laser on experimental orthodontic tooth movements with histomorphological methods.

## Methods

Dicle University Prof. Dr. Sabahattin Payzın Health Sciences Research and Application Center Experimental Animals Local Ethics Committee (DÜHADEK) received ethical approval on 13.05.2015 and this study was carried out in the animal laboratories of this center. Histological evaluation was done in Dicle University Faculty of Medicine, Department of Histology and Embryology.

### Animals

In the study, 30 12-week-old male *Rattus norvegicus albinus* Wistar were divided into three study groups with equal numbers (n = 10) (Table 1).

**Table 1:** Laser application time and applied force weight to all groups.

No		Groups		Number of subjects (n)		Force		Duration of laser application		Duration of the experiment
1		Group 1		10		20 g		12 min 72 J (7 days)		8 days
2		Group 2		10		20 g		9 min 54 J (7 days)		8 days
3		Control		10		20 g		----		8 days

Group 1: Laser was applied to the distovestibular, distal and distopalatinal alveolar regions of the upper right incisors for 12 min (720 s × 0.1 W = 72 J) for 7 days.

Group 2: Laser radiation was applied to the distovestibular, distal and distopalatinal alveolar region of the upper right incisors for 9 min (540 s × 0.1 W = 54 J) in 7 days.

Control Group: Laser radiation was not applied to the experimental.

### Making coil spring and its application

The active and passive tooth apparatus were designed and applied to obtain experimental orthodontic tooth movement in this study^35^.

**Figure 1:**
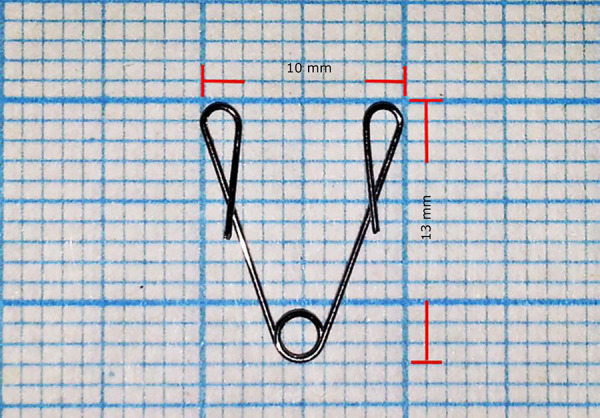
Passive configuration of appliance.

**Figure 2:**
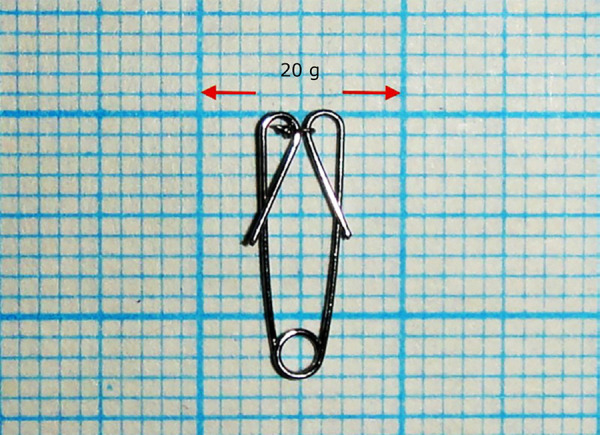
Active configuration of appliance.

The opening coil was completed by making a single helix with the second round (T2) of the Tweed plier and twisting by the same plier’s first round (T1) from 0.012-inch orthodontic wire (G&H straight lengths) at the figure that pre-prepared and drawn on millimeter paper. Appliance is prepared in this way to apply 20 g force when its inner parts are touched together. Active and passive views of the apparatus are shown in Figs. 1 and 2.

**Figure 3:**
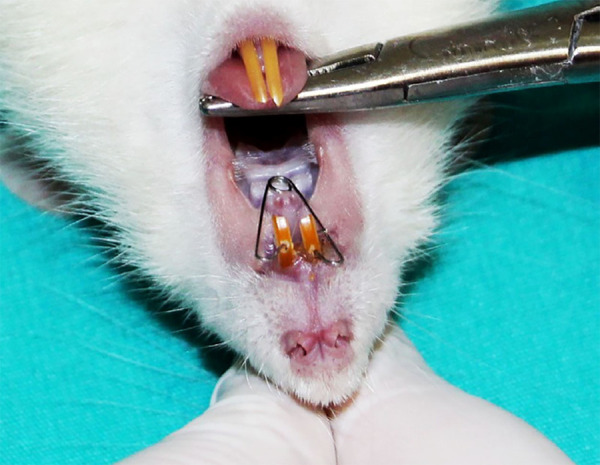
Intraoral view of the appliance applied to the experimental animal.

A hole was drilled with a bur in the vestibulopalatinal direction, 1.5 mm far from the gingiva, under local anesthesia provided with Ketas (90 mg/kg) and Xylazine (3 mg/kg), in order to ensure the retention of the appliance. A coil spring was applied, providing 20 g of force on all subjects’ (n = 30) teeth (Fig. 3).

Experimental animals were housed in a room that was dim in daytime and dark at night, in room temperature during the experiment. The feeds were given by wetting in order to prevent damage to the appliance and teeth. Weight tracking was performed for 8 days in order to evaluate weight changes. The gap between the upper incisors teeth of all experimental animals was measured with a digital caliper with a precision of 0.01 mm, 1.5 mm far from the gingiva, every day for 7 days after the application of the appliance. Measurements were made by one person.

### Diode laser application phase

Diode laser application was made with the 980 nm Smart 980-5 model of DEKA brand [DEKA M.E.L.A Srl via Baldanzese 17 500041 Calenzano (FI) Italy].

After application of the appliance, 72 J laser was applied to the first experiment group and 54 J laser was applied to the second experiment group for 7 days under general anesthesia. Laser fiber tip was in contact with mucous membrane. No laser was applied to the control group. During the laser application, protective glasses were worn against the harmful effects of the light and no one else was kept in the room at the time.

Laser was applied totally 12 min to the alveolar region of the right upper incisor of the 72 J group; in this way, distovestibular region 4 min, distal region 4 min, distopalatinal region 4 min.

Laser was applied totally 9 min to the alveolar region of the right upper incisor of the 54 J group; in this way, distovestibular region 3 min, distal region 3 min, distopalatinal region 3 min.

Experimental groups were not applied on the eighth day and all groups were sacrificed.

### Histopathological examination

Alveolar bone samples taken from the premaxillas of all three groups were stained with Hematoxylin-Eosin, Masson Trichrome and Periodic acid-Schiff (PAS). Serial sections were compared in terms of osteoblastic and osteoclastic activities.

The premaxilla samples of the subjects were kept in a 10% neutral formalin solution. Nitric acid of 2% was used for decalcification. 5 μm thick sections were obtained after reaching the tissues from paraffin blocks (Leica RM2265 Germany).

Sections painted were examined under the light microscope ZEISS (Carl Zeiss Microscopy GmbH Königsallee 9-21 37081 Göttingen, Germany) for histological evaluation and photos were taken with Zeiss Axio glass Imager A2 camera and obtained as ×100.

### Statistical evaluation

In this study, the suitability of the data to the normal distribution assumption was examined with the Kolmogorov-Smirnow test and the homogeneity was examined with the Levene test.

One Way Anova (ANOVA) test was used to determine the difference between the averages among the groups, Tukey-HSD and Dunnet-t multiple comparison tests were used to determine from which groups the difference originated from when it was statistically significant.

A 95% confidence interval was applied in statistical analysis tests. Descriptive statistics and analyzes were performed using the R version 3.2.3 (2015-12-10), Copyright (C) 2015 The R Foundation for Statistical Computing free software computer package program. The results were considered statistically significant for p < 0.05. [p < 0.05 (*), p < 0.01, (**), P > 0.05 n.s.].

## Results

### Observational findings

The subjects were weighed and their soft tissues were checked before applying laser every day. On the 2nd and 4th day of the experiment, one experimental animal from the control group died. On the 4th day of the experiment, an experimental animal from the 1st group, 72 J laser energy applied, also appeared to be displaying appliance due to the breakage of the tooth and the subject was excluded from the study.

### Weight changes

In the evaluation of weight change findings of all three groups, it was seen that the average weight loss was not statistically significant on any consecutive day. Only the first group (72 J) showed that the difference between the first day and the last day weight averages were statistically significant. Except for the first group (72 J), there was no significant statistical difference in weight loss between days in the other two groups.

### Orthodontic tooth movement changes

Statistical evaluation results of multiple comparison tests between days for groups are given in Table 2.

**Table 2:** Statistical evaluation results of multiple comparison tests between days for groups are given in the table (*p < 0.05, **p < 0.01).

Groups		Days		Days		p
72 J		2		3		**
				4		**
				5		**
				6		**
				7		**
				8		**
						
54 J		2		3		*
				4		**
				5		**
				6		**
				7		**
				8		**
						
Control		2		3		*
				4		*
				5		*
				6		*
				7		*
				8		*

Statistical evaluation results of multiple comparison tests of average tooth movements between groups for days are given in Table 3.

**Table 3:** Statistical evaluation results of multiple comparison tests of average tooth movements between groups for days are given in the table (*p < 0.05, ns: non-significant).

Days		Groups		Groups		p
2		72 J		54 J		ns
		72 J		Control		ns
		54 J		Control		ns
						
3		72 J		54 J		ns.
		72 J		Control		ns
		54 J		Control		ns
						
4		72 J		54 J		ns
		72 J		Control		ns
		54 J		Control		ns
						
5		72 J		54 J		ns
		72 J		Control		ns
		54 J		Control		*
						
6		72 J		54 J		ns
		72 J		Control		ns
		54 J		Control		*
						
7		72 J		54 J		ns
		72 J		Control		ns
		54 J		Control		ns
						
8		72 J		54 J		ns
		72 J		Control		ns
		54 J		Control		ns

### Histopathological findings

#### Histopathological findings of the control group

**Figure 4:**
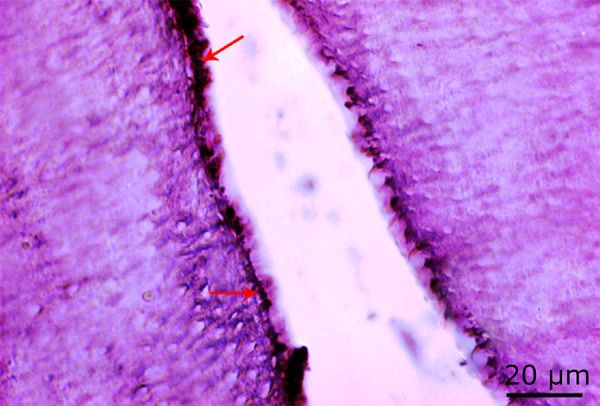
Normal osteoblast activity (red arrow) in the control group (H&E, ×100).

**Figure 5:**
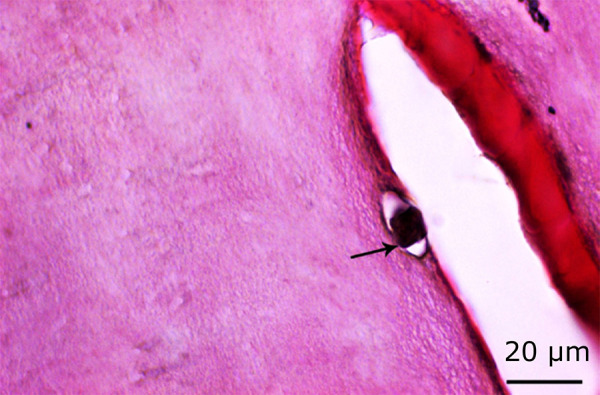
Normal osteoclast activity (*black arrow*) in the control group (H&E, ×100).

Osteoblastic and osteoclastic activity were observed to be normal in the alveolar bone sections of the control group (Figs. 4 and 5).

#### Histopathological findings of the 54 J laser group

**Figure 6:**
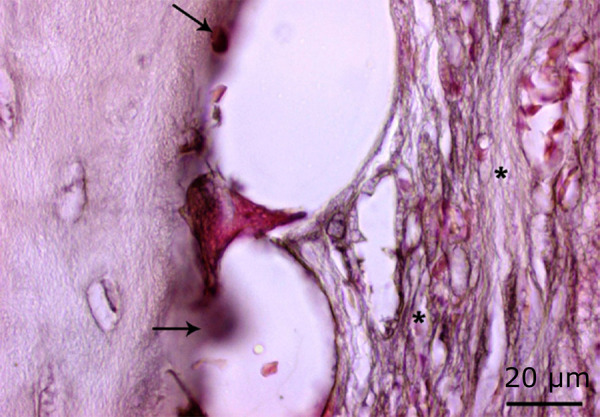
Increased osteoclast activity in the 54 J laser group (*black arrow*) and regions where bone destruction is intense (*) (H&E, ×100).

**Figure 7:**
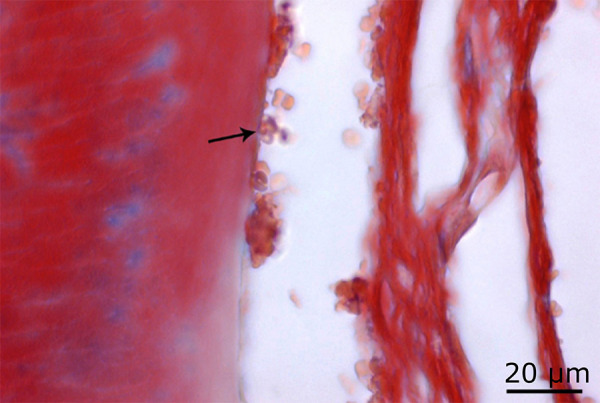
Decrease in osteoblast activity in the 54 J laser group (*black arrow*) (Masson’s trichrome staining, ×100).

**Figure 8:**
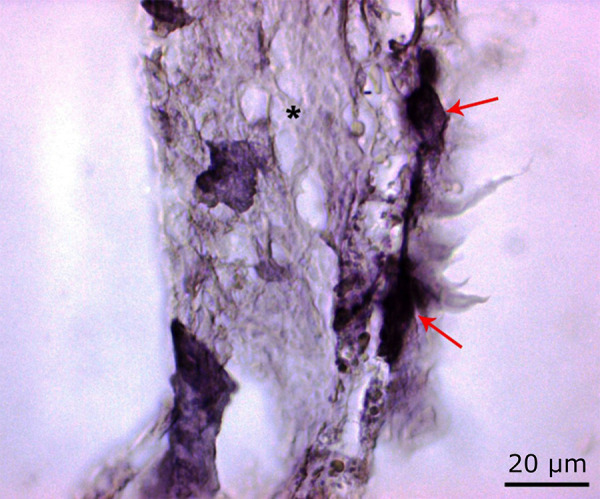
Increase in osteoclast activity in the 54 J laser group (*red arrow*) and density in the areas of bone degeneration (*) (PAS, ×100).

It was observed that osteblastic activity decreased in the sections belonging to the 54 J group, whereas osteoclastic activity increased (Figs. 6 to 8).

Histopathological findings of the laser group

**Figure 9:**
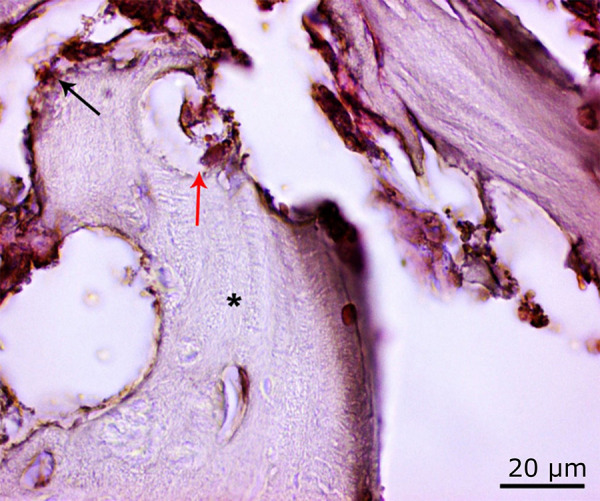
Osteoclast activity (*red arrow*) in the 72 J laser group. Osteoblastic activity (*black arrow*), which showed an increase when compared to the 54 J group and decrease when compared to the control group. Also, partial normalization in bone degeneration (*) (H&E, ×100).

**Figure 10:**
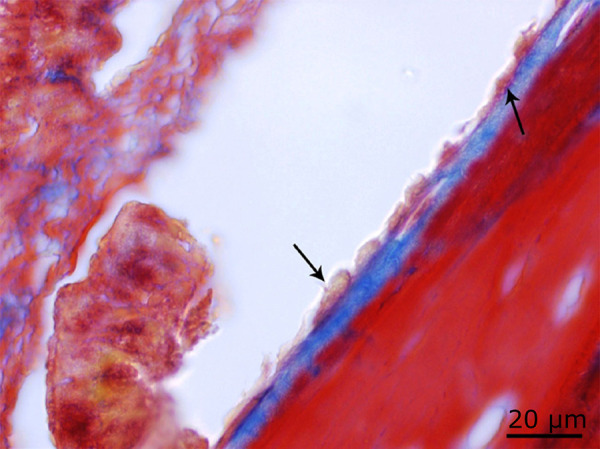
Increased osteoblastic activity in the 72 J laser group when compared to the 54 J group (*black arrow*) (Masson’s trichrome staining ×100).

**Figure 11:**
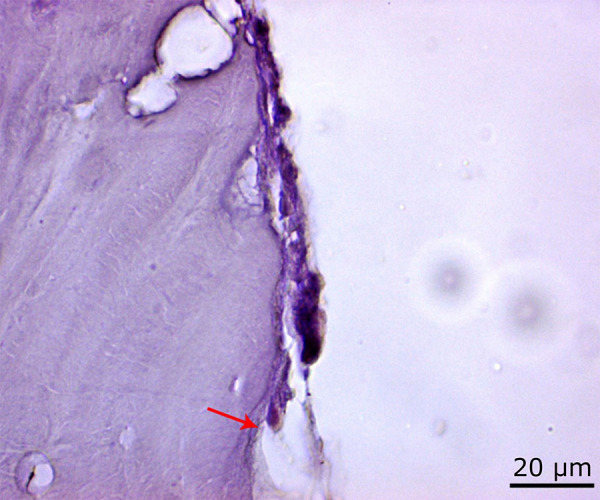
Increased osteoclastic activity in the 72 J laser group compared to the control group (*red arrow*) (PAS, ×100).

It was found that osteoclast activation increased in the 72 J group compared to the control group and decreased in the 72 J group compared to the 54 J group. (Figs. 9 to 11).

## Discussion

The aim of this study was to evaluate the effect of low-dose diode laser, which is a noninvasive method, on experimental orthodontic tooth movements in metric and histological way. Orthodontic strength was applied using a force element with the best application properties: high retention, stable, will prevent food retention and physiological functions, will not damage the surrounding tissues, an additional ligature wire will not be required as a holding element, so that the applied force can be easily removed and reinstalled when desired.

In previous years, many studies based on accelerating orthodontic tooth movement have been carried out and similar studies are still being carried out by researchers today. Studies evaluated in the three groups were chemical applications, surgical applications and mechanical-physical applications. Agents such as acetyl salicylic acid^35^, 1,25-dihydroxycholecalciferol^7^–^8^, nitric oxide^9^, thyroid hormones^10^, and cortisone^11^, as well as prostaglandins have been used in the type of studies^12^. As surgical applications, corticotomy and similar applications are among the studies carried out to accelerate the orthodontic tooth movement. In some experimental studies, in addition to the applied forces, mechanical vibration^15^, direct electrical current and pulsed electromagnetic physical applications have attempted to increase the speed of orthodontic tooth movement^16^–^17^. Although various local or systemic agents, such as inflammatory cytokines^13^, osteocalcin^14^, in order to accelerate orthodontic tooth movement, give promising results in experimental animals and in the laboratouar, Note that mucosal injections are clinically painful and may not be tolerated by patients.

In studies on experimental orthodontic tooth movement, rats were generally preferred because they can be produced and provided in a short period of time^36^. According to some studies, it has been reported that the biological response to the mechanical stimuli in periodontal ligament and alveolar bone in rats is very similar to that of humans at the cellular level^37^^,^ 38. For these reasons, it was important to use rats as experimental animals. Researchers stated that orthodontic tooth movements occur faster in young experimental animals^39^^,^ 40. In addition, it has been reported that tooth movement is slower in adults due to low proliferative activity in periodontal ligament and alveolar bone proportional to age^41^. After 2000, the effects of low-dose laser in experimental orthodontic tooth movements were examined in most studies, 6-12 weeks old rats were used^20^^,^ 24–29. In the studies, subjects may be excluded from the experiment for various reasons.

In the literature review, it was observed that molars^19^^,^ 20 ^,^ 23 ^,^ 24 ^,^ 26–29 ^,^ 42 ^,^ 43 and incisors^7^^,^ 25 ^,^ 35 ^,^ 44–46 were used for experimental orthodontic tooth movement. However, Altan *et al*.^25^ used only incisors in rat studies in which low-dose laser was examined for experimental orthodontic tooth movements. Considering the size of the oral structures and the oral cavities of the rats, it was decided to use incisors, considering that the application of the mechanics to the anterior teeth was less traumatic than the application to the posterior teeth.

There is no exact information about the optimum force level that will create experimental orthodontic tooth movement. Opinions are very different about the optimal force magnitude that can effectively move the teeth and act without causing tissue damage. Hermanson stated that the concept of “optimum force” will change according to individual difference^47^. Experimental tooth movement occured in the literature by applying force between 0.5 and 560 4 g^4^^,^ 46 ^,^ 48 ^,^ 49. Bister and Meikle^6^ stated that weak forces applied for the purpose of orthodontic tooth movement resorption in the alveolar bone and heavy forces caused the formation of hyalinization tissue.

Mitsui *et al*.^48^, in their invitro study, determined that the strength of 1 g/cm^2^ for human osteoblasts compared the effect of the forces between 0.5 and 3 g/cm^2^ revealed maximum PGE2 release^48^. Storey reported that there was no difference between 25 and 100 g force in terms of tissue response, but significant tissuel response difference occurred between 5 and 35 g forces^44^. The same researcher stated that it was a mistake to have more rapid tooth movements obtained with heavy forces in rats because this movement was due to the separation of the premaxillary segments^50^. In the light of this information, taking into account the past studies^7^^,^ 25 ^,^ 35 to effectuate experimental orthodontic tooth movement, That’s why it was decided to apply 20 g of force to provide pure tooth movement without creating hyalinized zones in the periodontal ligament and causing separation in the premaxilla.

There are important differences between animal studies in terms of duration of experiments and there are studies ranging from a few minutes to several months^37^^,^ 39 ^,^ 51–54. Bridges *et al*.^39^ examined that phases of tooth movements, which applied orthodontic force of 60 g to 3-4 rats weighing 50-60 g and 28 rats of 12-13 weeks weighing 300-500 g and the effects of age on orthodontic tooth movements, and reported that the cycle of tooth movements consisted of three phases. They reported that in the first phase, due to the viscoelastic structure of the tissues, there was an instant tooth movement. In the second phase (delay period), as a result of hyalinization and indirect bone resorption, minimal tooth movement occurred, and in the third phase (late period) “remodeling” and orthodontic tooth movement occurred. In the same study, they reported that the tooth movement cycle in rats was 7 days^39^. Marquezan *et al*.^28^ examined the effects of low-dose laser on experimental orthodontic dental movement in their study, the duration of the experiments as 2 days and 7 days. According to the results, they reported that there was a significant increase number of osteoclastic cells in the 7-day laser treated rats, although the results are not statistically significant. In the light of this information, the duration of the study was determined as 8 days, in order to see the effect of the low-dose laser applied to the orthodontic tooth movement that occurs by adapting the appliance to occur pure orthodontic tooth movement. By using the biostimulant effect of low-dose laser, it was determined to accelerate orthodontic tooth movement and at which energy dose biostimulation was more effective.

Laser devices are preferred according to the tissue to be studied and the desired effect. Some authors have stated that the optimal wavelength for biostimulation is between 550-970 nm^55^^,^ 56. They argued that wavelengths above this range would be absorbed by the superficial layers of tissue and the deep tissues would not be stimulated^57^. The wavelength of the laser device used in this study is 980 nm. This value is close to the specified wavelength range and there has been an increase in osteoclastic activity that stimulated bone destruction cells under soft tissue in laser treated areas.

In animal studies examining the effects of a low dose laser on orthodontic tooth movement, the power of the laser used is 5-100 mW^1^^,^ 19–21 ^,^ 23 ^,^ 25 ^,^ 26 ^,^ 42 ^,^ 43 ^,^ 58 ^,^ 59. Some researchers may report that a laser with an output power of 1-2 mW can also achieve biostimulant effects; however, they stated that it was much easier to achieve the same effects with a laser 100 times stronger^60^. Equivalent energy can be applied by changing the duration of treatment according to the power of the laser. However, as it is thought that sufficient energy can be transferred deeper into the tissue as the power is increased, the treatment prolongation cannot compensate for the low power intensity^60^^,^ 61. In the light of this information, The laser device is set to 100 mW of power, taking into account the latest studies^24^–^26^^,^ 28 ^,^ 43.

There are several animal studies on the effects of the low dose laser on experimental orthodontic tooth movement. Rats were used as experimental animals in nine of them^19^^,^ 20 ^,^ 23–29. The first of these studies was done by Kawasaki and Shimizu^19^. While continious laser beam is used in most studies, Duan *et al*.^29^ examined the differences between continious and interrupted laser beam application and that both produced faster orthodontic tooth movement; however, they reported that there were no significant differences between the lasers. Unlike most of the studies, Altan *et al*.^25^, Gama *et al*.^27^ and Marquezan *et al*.^28^ reported that the effect of low-dose laser on experimental orthodontic tooth movement speed did not differ significantly from control groups. However, in the remaining 6 studies, the low-dose laser was reported to accelerate experimental orthodontic tooth movement with a statistically significant difference. Torri and Weber^62^ reported that, in their review, when they evaluated the effects of low-dose laser therapies on orthodontic dental movements, the most used and most effective dose was 54 J in animal studies and 2 J dose in human studies^62^.

The mechanism of action of low-dose laser therapies work with the capability of subcellular photoreceptors to respond to visible red and infrared wavelengths. With the stimulation of these receptors, changes occur in the enzymatic and photochemical activities of tissues^30^^,^ 31. Electron transport chain, respiratory cycle and oxidation mechanisms are affected and an increase in cellular metabolic processes occur^32^. At the same time, effects such as increase in fibroblastic activities (fibroblast proliferation, collagen secretion, collagen matrix deposition), increase in osteoblastic and osteoclastic activities and acceleration of bone resorption and the alveolar bone cycle have been shown^30^^,^ 33 ^,^ 34. In the light of these histomorphological effects, the important issue in histopathological evaluation is the osteoblastic activity occurring in the alveolar bones due to experimental orthodontic tooth movement. In histopathological examination of tissue samples, the preparations that emerged were compared and it was observed that osteoblastic activity in the third group was normal. However, it was also observed that groups 1 and 2 (laser groups) had less osteoblastic activity than the control group. This is incompatible with some researchers findings that low-dose laser energy has a stimulative effect on osteoblastic activity^25^^,^ 61 ^,^ 63–65. According to our findings, a decrease in osteoblastic activity in the 54 J group (group 2) was observed more than in the 72 J (group 1) group. When the teeth are moved in the bone, resorption occurs on the bone surface adjacent to the periodontal ligament. In this case, the compatibility of the osteoclastic activity in the pressure zone with the osteoblastic activity in the tension zone provides a “remodeling” cycle similar to that in the physiological tooth movement. In this study, the alveolar bone in the direction of movement was examined (pressure zone). Therefore, it is expected that the osteoblastic activity (which is normally accepted) in the third group is actually lower than the stretching zone, according to the physiological tooth movement principles. In this case, in order to fully evaluate the effects of low-dose laser on osteoblasts in experimental orthodontic tooth movements, it is necessary to take a sample from the tension zone and evaluate it in more detail.

In the evaluation of histopathological findings of the alveolar bone, it was observed that osteoclastic activity increased in the second group and osteoblastic activity decreased compared to other groups. On the other hand, it was determined that osteoclastic activity increased in the first group, but this increase was less than that in the second group and more than the control group. Finally, an increase in osteoclastic activity in the alveolar bone in the direction of movement was observed in the control group, but this increase was found to be less than that of the laser applied groups. According to the results obtained, the observation that there was an increase in osteoclast activity in the second group compared to the other groups and the osteoclast activity in the first group compared to the control group shows that low-dose laser energy stimulates osteoclastic activity from the early stages of dental movement. These findings are consistent with studies reporting that laser application increases multinucleated osteoclast cells^19^^,^ 20 ^,^ 23 ^,^ 25 ^,^ 42 ^,^ 43. Depending on orthodontic tooth movements, it was determined that the amount of tooth movement between the groups was higher in places where osteoclastic activity was high. It is compatible with the prediction that there will be more resorption in the regions where osteoclastic activity is high and, therefore, more tooth movement will occur.

## Conclusions

It was found effective to apply 54 J laser energy to increase the amount of orthodontic tooth movement; however, 72 J laser energy obtained by increasing the dose was not seen as effective. Accordingly, the use of diode laser (low-dose laser) in experimental orthodontic tooth movement increases the amount of tooth movement; however, it has been concluded that this situation depends on the dose administered and studies on this subject are needed.
